# An audit of the medical pre-clinical curriculum at an urban university: sexual and gender minority health content

**DOI:** 10.1080/10872981.2021.1947172

**Published:** 2021-07-02

**Authors:** Mandi L. Pratt-Chapman, Nina Abon

**Affiliations:** School of Medicine and Health Sciences, The George Washington University, Washington, DC

**Keywords:** LGBTQI, sexual and gender minority, curriculum, audit

## Abstract

Most medical students receive inadequate preparation to care for sexual and gender minority (SGM) patients. A review of one urban medical school’s pre-clinical curriculum was conducted to assess coverage of appropriate SGM health content. Curricula that fully or partially addressed American Association of Medical Colleges (AAMC) core competencies for SGM health were categorized in an Excel spreadsheet. For partially met competencies, content that addressed the competency along with what was needed to fully address the competency were documented. AAMC SGM competencies that were not addressed at all were also noted. As a secondary source for triangulation, curricular topics were compared to SGM health content prioritized by Vanderbilt, a leader in championing inclusion of SGM content in medical curricula. Of the 30 AAMC competencies, 10 competencies were addressed, 11 were partially addressed, and 9 were not addressed. Gaps were noted in the AAMC domains of professionalism, systems-based practice, interprofessional collaboration, and personal/professional development. Among Vanderbilt topics, the George Washington University (GW) curriculum lacked content in intersex health, sexually transmitted infections (STIs) in lesbians, vaginitis in lesbians, efficacy of anal microbicides, anal Pap smears, and anal cancer risk and treatment for men who have sex with men (MSM). Despite these weaknesses, GW clocked greater than the national average at 7.5 hours of SGM content. This study provides a roadmap for curricular enhancements needed at GW as well as a prototype for other institutions to audit and improve curricular coverage on SGM health.

## Introduction

Health disparities in the lesbian, gay, bisexual, transgender, queer, and intersex (LGBTQI) communities have been well documented in recent years. Most well-known are the disproportionate rates of sexually transmitted infections (STIs) in men who have sex with men (MSM) and male-to-female (MtF) transgender patients[1]. In addition, there has been research documenting increased behavioral risks among sexual and gender minority (SGM) people, including higher smoking rates, obesity, depression and other mental health disorders, and mortality from certain cancers[1]. SGM individuals also show lower health-care utilization, in part due to perceived and real discrimination from health-care providers[1]. A national survey of transgender patients found that 19% had been refused care due to their gender identity or expression, 28% reported being verbally harassed in a medical setting, and one-fourth of survey participants had delayed needed care because of disrespect and discrimination from medical providers[2]. Reports have shown that SGM people often choose not to disclose their sexual orientation or gender identity to health-care providers due to actual or anticipated discrimination[3]. This fear is not unwarranted: A survey of medical students published in 2015 found that 46% expressed explicit bias against SGM and 82% had some form of implicit bias[4].

A 2011 study found that the median number of hours that a medical student in the US is exposed to SGM content is 5 hours[5]. The same study found that though students were taught to ask patients: ‘Do you sleep with men, women, or both?,’ students felt they were not given enough instruction on how to counsel patients on safer sex behaviors for those who reported same-sex sexual practices. Another study showed that most medical students rated their school’s curriculum as ‘fair’ or worse in preparing them to care for SGM patients[6]. Students reported feeling most prepared to address human immunodeficiency virus (HIV) and other sexually transmitted infections (STIs) and felt less prepared addressing sex reassignment surgery and gender transitioning for transgender and gender nonconforming patients[6].

In 2014, the Association of American Medical Colleges (AAMC) released their seminal publication, *Implementing Curricular and Institutional Climate Changes to Improve Health Care for Individuals Who Are LGBT, Gender Nonconforming, or Born with DSD: A Resource for Medical Educators*[7]. This publication offers guidance to implement 30 recommended competencies for affirming LGBTQI care. The present study sought to answer the question: To what degree does the George Washington University (GW) core medical curriculum address important SGM competencies and topics for medical students?

## Materials and methods

### Participants and setting

We conducted an audit of pre-clinical medical curricula since all students are exposed to this content. Clinical content varies based on clinical rotations and sites, and is unlikely to include SGM content unless it is specifically sought by the student.

### Study design

The authors compared the content to the AAMC competencies [7] and to learning objectives established by Vanderbilt University. Content was catalogued as fully, partially, or not addressing content based on a previously developed protocol[4].

### Data sources

Data sources included the curriculum database, the 2016–2017 Clinical Skills and Reasoning (CSR) manual, professional development session notes, lecture notes from one student (NA), and student feedback on the accuracy of findings. Faculty feedback provided additional information on curricular changes after 2017.

### Procedures

A medical student (NA) reviewed content from lectures and searched the curriculum database for learning objectives that included keywords: lesbian, gay, bisexual, trans, LGB, GLB, LGBT, MSM, WSW, MTF, FTM, homosexual, intersex, sex development, DSD, sexual orientation, and gender dysphoria. The database generated a report including the course title, block, and session objectives. The CSR manual was examined for keywords and topics. NA assessed professional development sessions and preclinical lectures by reviewing personal notes from coursework.

A matrix was compiled that displayed each AAMC competency domain and competency statement along with sessions that addressed the competency, format, and specific examples (see Table 1). AAMC competency domains mirror the recommendations of the Accreditation Council for Graduate Medical Education/American Board of Medical Specialties; however, the competency statements under each domain illustrate competence in specific SGM health areas that medical students should be aware of, able to assess, and/or able to manage clinically[8]. A separate table (Table 3) compares content with Vanderbilt learning objectives.

**Table 1.** Medical school curricular alignment with association of American medical colleges competencies to improve health care for LGBTQI individuals

	Met	Partially met	Not met
**Competency Domain: Patient Care** *Gather essential and accurate information about patients and their conditions through history taking, physical examination, and the use of laboratory data, imaging, and other tests by:*			
1. Sensitively and effectively eliciting relevant information about sex anatomy, sex development, sexual behavior, sexual history, sexual orientation, sexual identity, and gender identity from all patients in a developmentally appropriate manner.	✓		
2. Performing a complete and accurate physical exam with sensitivity to issues specific to the individuals described above at stages across the lifespan. This includes knowing when particulars of the exam are essential and when they may be unnecessarily traumatizing (as may be the case, for example, with repeated genital exams by multiple providers).		✓	
*Make informed decisions about diagnostic and therapeutic interventions based on patient information and preferences, up-to-date scientific evidence, and clinical judgment by:*			
3. Describing the special health care needs and available options for quality care for transgender patients and for patients born with DSD (e.g., specialist counseling, pubertal suppression, elective and nonelective hormone therapies, elective and nonelective surgeries, etc.).		✓	
*Counsel and educate patients and their families to empower them to participate in their care and enable shared decision-making by:*			
4. Assessing unique needs and tailoring the physical exam and counseling and treatment recommendations to any of the individuals described above, taking into account any special needs, impairments, or disabilities.	✓		
5. Recognizing the unique health risks and challenges often encountered by the individuals described above, as well as their resources, and tailoring health messages and counseling efforts to boost resilience and reduce high-risk behaviors.	✓		
*Provide health care services to patients, families, and communities aimed at preventing health problems or maintaining health by:*			
6. Providing effective primary care and anticipatory guidance by utilizing screening tests, preventive interventions, and health care maintenance for the populations described above (e.g., screening all individuals for inter-partner violence and abuse; assessing suicide risk in all youth who are gender nonconforming and/or identify as gay, lesbian, bisexual and/or transgender; and conducting screenings for transgender patients as appropriate to each patient’s anatomical, physiological, and behavioral histories).		✓	
Competency Domain: Knowledge for Practice *Apply established and emerging biophysical scientific principles fundamental to health care for patients and populations by:*			
7. Defining and describing the differences among: sex and gender; gender expression and gender identity; gender discordance, gender nonconformity, and gender dysphoria; and sexual orientation, sexual identity, and sexual behavior.	✓		
8. Understanding typical (male and female) sex development and knowing the main etiologies of atypical sex development.	✓		
9. Understanding and explaining how stages of physical and identity development across the lifespan affect the above-described populations and how health care needs and clinical practice are affected by these processes.		✓	
*Apply principles of social-behavioral sciences to the provision of patient care, including assessment of the impact of psychosocial and cultural influences on health, disease, care seeking, care compliance, and barriers to and attitudes toward care by:*			
10. Understanding and describing historical, political, institutional, and sociocultural factors that may underlie health care disparities experienced by the populations described above.		✓	
*Demonstrate an investigatory and analytic approach to clinical situations by:*			
11. Recognizing the gaps in scientific knowledge (e.g., efficacy of various interventions for DSD in childhood; efficacy of various interventions for gender dysphoria in childhood) and identifying various harmful practices (e.g., historical practice of using ‘reparative’ therapy to attempt to change sexual orientation; withholding hormone therapy from transgender individuals) that perpetuate the health disparities for patients in the populations described above.		✓	
Competency Domain: Practice-Based Learning and Improvement *Identify strengths, deficiencies, and limits in one’s knowledge and expertise by:*			
12. Critically recognizing, assessing, and developing strategies to mitigate the inherent power imbalance between physician and patient or between physician and parent/guardian, and recognizing how this imbalance may negatively affect the clinical encounter and health care outcomes for the individuals described above.		✓	
13. Demonstrating the ability to elicit feedback from the individuals described above about their experience in health care systems and with practitioners, and identifying opportunities to incorporate this feedback as a means to improve care (e.g., modification of intake forms, providing access to single-stall, gender-neutral bathrooms, etc.).	✓		
*Locate, appraise, and assimilate evidence from scientific studies related to patients’ health problems by:*			
14. Identifying important clinical questions as they emerge in the context of caring for the individuals described above, and using technology to find evidence from scientific studies in the literature and/or existing clinical guidelines to inform clinical decision making and improve health outcomes.			✓
Competency Domain: Interpersonal and Communication Skills *Communicate effectively with patients, families, and the public, as appropriate, across a broad range of socioeconomic and cultural backgrounds by:*			
15. Developing rapport with all individuals (patient, families, and/or members of the health care team) regardless of others’ gender identities, gender expressions, body types, sexual identities, or sexual orientations, to promote respectful and affirming interpersonal exchanges, including by staying current with evolving terminology.	✓		
16. Recognizing and respecting the sensitivity of certain clinical information pertaining to the care of the patient populations described above, and involving the patient (or the guardian of a pediatric patient) in the decision of when and how to communicate such information to others.			✓
*Demonstrate insight and understanding about emotions and human responses to emotions that allow one to develop and manage interpersonal interactions by:*			
17. Understanding that implicit (i.e., automatic or unconscious) bias and assumptions about sexuality, gender, and sex anatomy may adversely affect verbal, nonverbal, and/or written communication strategies involved in patient care, and engaging in effective corrective self-reflection processes to mitigate those effects.	✓		
18. Identifying communication patterns in the health care setting that may adversely affect care of the described populations, and learning to effectively address those situations in order to protect patients from the harmful effects of implicit bias or acts of discrimination.	✓		
Competency Domain: Professionalism *Demonstrate sensitivity and responsiveness to a diverse patient population, including but not limited to diversity in gender, age, culture, race, religion, disabilities, and sexual orientation by:*			
19. Recognizing and sensitively addressing all patients’ and families’ healing traditions and beliefs, including health-related beliefs, and understanding how these might shape reactions to diverse forms of sexuality, sexual behavior, sexual orientation, gender identity, gender expression, and sex development.			✓
20. Recognizing the unique aspects of confidentiality regarding gender, sex, and sexuality issues, especially for the patients described above, across the developmental spectrum, and by employing appropriate consent and assent practices.			
*Demonstrate accountability to patients, society, and the profession by:*			
21. Accepting shared responsibility for eliminating disparities, overt bias (e.g., discrimination), and developing policies and procedures that respect all patients’ rights to self-determination.			✓
22. Understanding and addressing the special challenges faced by health professionals who identify with one or more of the populations described above in order to advance a health care environment that promotes the use of policies that eliminate disparities (e.g., employee nondiscrimination policies, comprehensive domestic partner benefits, etc.).			✓
Competency Domain: Systems-Based Practice *Advocate for quality patient care and optimal patient care systems by:*			
23. Explaining and demonstrating how to navigate the special legal and policy issues (e.g., insurance limitations, lack of partner benefits, visitation and nondiscrimination policies, discrimination against children of same-sex parents, school bullying policies) encountered by the populations described above.			✓
*Coordinate patient care within the health care system relevant to one’s clinical specialty by:*			
24. Identifying and appropriately using special resources available to support the health of the individuals described above (e.g., targeted smoking cessation programs, substance abuse treatment, and psychological support).			✓
25. Identifying and partnering with community resources that provide support to the individuals described above (e.g., treatment centers, care providers, community activists, support groups, legal advocates) to help eliminate bias from health care and address community needs.			✓
*Participate in identifying system errors and implementing potential systems solutions by:*			
26. Explaining how homophobia, transphobia, heterosexism, and sexism affect health care inequalities, costs, and outcomes.	✓		
27. Describing strategies that can be used to enact reform within existing health care institutions to improve care to the populations described above, such as forming an LGBT support network, revising outdated nondiscrimination and employee benefits policies, developing dedicated care teams to work with patients who were born with DSD, etc.			✓
*Incorporate considerations of cost awareness and risk-benefit analysis in patient and/or population-based care by:*			
28. Demonstrating the ability to perform an appropriate risk/benefit analysis for interventions where evidence-based practice is lacking, such as when assisting families with children born with some forms of DSD, families with pre-pubertal gender nonconforming children, or families with pubertal gender nonconforming adolescents.		✓	
Competency Domain: Interprofessional Collaboration			
29. Work with other health professionals to establish and maintain a climate of mutual respect, dignity, diversity, ethical integrity, and trust by: Valuing the importance of interprofessional communication and collaboration in providing culturally competent, patient-centered care to the individuals described above and participating effectively as a member of an interdisciplinary health care team.		✓	
Competency Domain: Personal and Professional Development			
30. Practice flexibility and maturity in adjusting to change with the capacity to alter one’s behavior by: Critically recognizing, assessing, and developing strategies to mitigate one’s own implicit (i.e., automatic or unconscious) biases in providing care to the individuals described above and recognizing the contribution of bias to increased iatrogenic risk and health disparities.		✓

**Table 2.** Partially covered AAMC competencies

Competency	Covered	Not covered
2	Gender expression not equating to anatomy	Repeat genital exams by multiple providers traumatizing to intersex patients
3	Hormonal and surgical options for transgender individuals	Multidisciplinary specialty referral for transgender individuals
6	Cancer screening based on anatomy and hepatitis vaccinations for individuals who engage in anal sex	Indications for hormonal replacement among intersex patients
9	Adolescent transgender content was provided including anxiety or loneliness stemming from social isolation or family rejection	Counseling needs of families with intersex children
10	Medical pathology of homosexuality and laws that discriminate against same-sex benefits	Analyzing clinical practices to address the needs of intersex patients
11	Dangers of conversion therapy and mental health benefits of affirming-transgender care	Lack of evidence to support genital ‘normalization’ for intersex patients
12	Awareness of power imbalance between providers and genderqueer adolescents	Shared decision-making with parents of intersex children
20	Patient confidentiality and adolescent-specific concerns	Concerns and risks of disclosure of SGM patient information
28	Dangers of conversion therapy	Social transition for transgender youth with patients, weighing pros and cons of pubertal suppression for transgender youth, and assisting families with understanding the medical management implications for intersex patients
29	Working with a multidisciplinary team for appropriate transgender care	Working with a multidisciplinary team to address appropriate intersex care, bullying directed toward LGBTQI youth, substance abuse treatment for SGM, and care coordination
30	Recognizing that biology and identity do not always align	Self-reflection on assumptions about necessary care and suspending judgment about patient behavioral risks

**Table 3.** Medical school curricular alignment with Vanderbilt topics for LGBTQI health

SGM-Specific Health Topic	Addressed?	Explanation
Communication/Interview Skills	Yes	Addressed in ‘LGBTQ Health Basics’ in Foundations block and in ‘Sexual History Interviewing’ in Reproduction and Endocrinology block
Intake Forms (gender identity, sexual orientation, relationship status, parentage)	Yes	Discussed how to build a culture of respect and openness which included slides showing intake forms in ‘LGBTQ Health Basics’
Assumptions/Biases	Partially	The presence of assumptions and biases are discussed in lectures, but students are not given time to explore or reflect on their own biases specific to SGM populations.
Depression Screening	Yes	Addressed increased prevalence of depression in LGBTQ population in ‘LGBTQ Health Basics.’ Depression screening taught in Brain and Behavior block.
Substance Abuse Screening	Yes	Addressed increased prevalence of substance abuse in ‘LGBTQ Health Basics.’ Substance use disorder screening taught in Brain and Behavior block.
SGM Standardized Patient Cases	Partially	There are five lesbian or gay standardized patient scenarios and one bisexual patient scenario throughout the curriculum (bisexual patient newly added). There are no transgender, gender-nonconforming, nor intersex patient scenarios. Two transgender cases have subsequently been developed for fellows and practitioners, but they are not integrated with student curricula.
PBL (Problem Based Learning) Integration	Yes	Addressed by use of lesbian or gay standardized patients and in Clinical Public Health Summit on HIV/AIDS
Embryology: Disorders of Sex Development	Partially	‘Mechanisms of Sex Determination’ lecture in Reproduction and Endocrinology block. However, this lecture was taught by research faculty and focused on physiology. Lacked discussion on clinical care and interdisciplinary teams needed to care for intersex patients.
Embryology: Gender vs. Sex	Yes	Presentations in ‘LGBTQ Health Basics’ and various lectures in Reproduction and Endocrinology block
Embryology: Changing Terminology	Yes	Presentations in ‘LGBTQ Health Basics’ and various lectures in Reproduction and Endocrinology block
Infectious Disease: Sexually Transmitted Infections in in lesbians	No	Was not in curriculum prior to 2018, but has been added.
Infectious Disease: Vaginitis in lesbians	No	Not covered.
Infectious Disease: STI recommendations in MSM	Partially	Guidelines in pre-session readings in ‘Introduction to Cultural Competency,’ but not discussed in lecture
HIV in Men who have Sex with Men (MSM)	Yes	HIV-specific lectures in Immunohematology and Infection block and in Clinical Public Health Summit on HIV/AIDS
Availability/ efficacy of rectal microbicides	No	Not covered.
Exclusive Women who have Sex with Women (WSW): Pap, Breast Exams, HPV screening	Yes	Addressed in LGTBQ Healthcare 1 lecture.
Anal Paps	No	Not covered.
MSMs and need of Hep A/ HPV shot	Yes	‘Infectious Causes of Liver Disease’ lecture in Immunohematology and Infection block
Hormone Therapy Pharmacology	Yes	Readings and lecture for ‘Transgender Medicine’ in Reproduction and Endocrinology block
Transitioning Options and Associated Risks	Yes	Readings and lecture for ‘Transgender Medicine’ in Reproduction and Endocrinology block
Puberty suppression in trans youth	Yes	Readings and lecture for ‘Transgender Medicine’ in Reproduction and Endocrinology block
Lesbian obesity	Yes	Readings and lecture for ‘Transgender Medicine’ in Reproduction and Endocrinology block
Increased heart disease rate in lesbians	Yes	Addressed in LGTBQ Healthcare 1 lecture
Anal cancer, risk, treatment in MSM	No	Not covered.
Lesbian nulliparity and risk of breast/ ovarian/ cervical cancer	Yes	Addressed in LGTBQ Healthcare 1 lecture
Psychological/ sexual/ coming out/ identity development	Yes	Discussed in ‘LGBT Health Basics’ and ‘Transgender Medicine’
Gay couples and fertility options	Yes	Focus of LGBQ Health II during Reproduction and Endocrinology block with panel including gay/lesbian parents and a Reproductive Endocrinology and Infertility clinician
Gender dysphoria vs. transgender	Yes	Discussed in various lectures- LGBTQ Health Basics (Foundations block), Brain and Sex (Brain and Behavior block), and Transgender Medicine (Reproduction and Endocrinology block)
Depression and suicide rates in LGBTQI teens/adults	Yes	Discussed in ‘Intro to Cultural Competency’ and ‘LGBT Health Basics’ lectures in Foundations block
Eating disorders in MSM	Partially	In ‘Eating Disorders’ (GI-Liver block) pre-session readings, but not in lecture
LGBTQI patients and having children (medical options and legal concerns)	Yes	Focus of ‘LGBQ Health’ during Reproduction and Endocrinology block with panel including gay/lesbian parents and a Reproductive Endocrinology and Infertility clinician
LGBT Teen Issues	Yes	Covered in sexually active adolescent panel discussion.

Member checking by four additional students and one faculty member confirmed the findings.

## Results

The pre-clinical curriculum met 10 of the 30 AAMC competencies (see Table 1). Of the eight AAMC competency domains, the most competencies fully addressed fell under the domains of patient care, knowledge for practice, practice-based learning, and interpersonal/communication skills. For example, within the patient care domain, the curriculum adequately addressed content to help students sensitively elicit relevant information about sex anatomy, sex development, sexual behavior, sexual history, sexual orientation, sexual identity, and gender identity and also covered assessing unique health risks and tailoring physical exams, counseling, and treatment recommendations for SGM patients. Within the knowledge for practice domain, the curriculum appropriate included content on 1) the differences between sex and gender; gender expression and identity; gender discordance v. nonconformity v. dysphoria; and sexual orientation, identity and behavior; and 2) typical sex development and etiologies of atypical development. Within the interpersonal and communication skills domain, the development of rapport with diverse SGM patients was adequately covered. For documentation of all fully addressed competencies, see Table 1.

Examples of competencies not at all met included: 1) identifying important clinical questions specific to SGM patients and finding evidence from research to inform clinical decision-making; 2) respecting the sensitivity of clinical information and considerations of when and how to communicate information about SGM status to others; 3) awareness of how SGM status might affect health-related beliefs; 4) acceptance of shared responsibility for eliminating bias in healthcare; 5) understanding the challenges faced by SGM health-care professionals; 6) navigating legal and policy challenges affecting the health and health care of SGM persons; 7) identifying SGM-affirming resources to support behavioral health; 8) partnering with community-based organizations to eliminate bias from health care and support SGM patient health; and 9) identifying strategies for reform to improve SGM health care. For documentation of all competencies not at all addressed, see Table 1.

For competencies partially met, most often intersex content was missing. An example of a partially met competency was the performance of a complete, accurate and sensitive physical exam across the SGM patient lifespan. While the content included an explanation of the difference between gender expression and anatomy, awareness that repeat genital exams by multiple providers could be traumatizing – particularly for intersex patients – was not covered. For a full description of partially met competencies along with what content was covered and what was not, see Table 2.

Of Vanderbilt topics, 22 were addressed, 5 were partially addressed, and 5 were not addressed. Curricula lacked content on STIs and vaginitis in lesbians, efficacy of anal microbicides, anal Pap smears, increased heart disease in lesbians, anal cancer risk in men who have sex with men (MSM), and lesbian nulliparity and cancer risk. Exploration of bias was done in professional development sessions, but how bias affects SGM patients specifically was not addressed. Small group sessions included two cases and five standardized patient scenarios which portrayed SGM patients, most of which involved gay men with HIV.

## Discussion

### Restatement of key findings

Overall, 28 sessions were found to include relevant material specific to SGM patients. Three sessions (LGBTQ Health Basics, Transgender Medicine, and LGBQ Health) focused specifically on SGM topics. These three sessions were mandatory and totaled 7.5 hours. A strength of the current curriculum was the opportunity for students to interact with the SGM community. While patient panels were well rated by students, some students expressed a desire for greater diversity among volunteers who were mostly white and of higher socioeconomic status. This critique reflects a lack of intersectionality in the curriculum. Gaps in addressing AAMC competency domains primarily included areas of professionalism, systems-based practice, interprofessional collaboration, and personal and professional development.

### Comparison to other findings

This curricular audit indicated that GW’s medical school curricula met more AAMC competencies for SGM health care than some other institutions that have conducted similar assessments and but less than others who have taken a leadership role in implementing SGM health-care curricula. For example, GW met 10 AAMC competencies fully compared to 7 fully met by Georgetown’s medical school curricula[9]. Yet, institutions, such as the University of Louisville, Kentucky [10,11] and the University of California Davis [12] have made greater strides in addressing medical student competence for serving SGM patients by taking a systems approach to reform of curricula.

### Explanation of differences among findings

A recent review of institutions leading SGM curricular change in health-care professional schools noted that empowered, motivated champions for change; available content expertise; alignment with organizational culture; institutional commitment; and inclusive strategic planning were keys to successful SGM health curricular change[13]. A key lesson learned from that review was ensuring that responsibility for inclusion of SGM health content is spread across faculty, not dependent on one or few faculties [13]. Medical schools that wish to bolster SGM health content can leverage these lessons learned to facilitate change.

### Limitations

This study was limited to a review of the preclinical curriculum at one point in time at one institution. Only one medical student conducted the review. The search terms used do not reflect the full spectrum of terms that SGM individuals use to identify themselves.

### Strengths

This study is among the earliest systematic assessments of a medical school curriculum in regard to AAMC-endorsed medical student competencies for SGM health. The triangulation of data from the curriculum database, student notes, and faculty and review of findings are a strength of the study.

### Next steps

Recommendations to address unmet competencies include diversifying standardized patients, case vignettes, and group discussions. More content on lesbian, bisexual, transgender, and intersex health needs; physician roles in challenging policies that perpetuate SGM inequities; and system-level strategies to provide more affirming SGM health care are warranted. After this assessment, clinical cases and standardized patient scenarios which portrayed SGM patients were expanded to include a lesbian woman seeking a referral for family planning, an elderly woman grieving over the loss of a female partner, a same-sex couple in the emergency room with suspected domestic violence, and a young man visiting a new provider who expresses shame regarding his attraction to other men. Additionally, an introduction of lesbian STIs and a bisexual standardized patient case has been added.

### Resources

Resources available to improve SGM content in medical schools include the AAMC publication of core competencies for providing affirming SGM health care[7], the Med Ed Portal[14], the National LGBT Health Education Center[15], the GW Cancer Center[16], the University of California San Francisco Center of Transgender Excellence[17], the World Professional Association for Transgender Health (WPATH)[18], the Endocrine Society[19], and InterAct and Lambda Legal[20].

## Conclusions

A growing awareness of the health risks and health-care needs of SGM individuals demands responsive curricula from medical schools. This study models a systematic way to identify gaps to target curricular enhancements in medical education and training – specifically by applying new curricular standards and by leveraging insights from a student investigator as an experiential researcher.

Overall, this study suggests the need for thoughtful integration of content into the medical school curriculum to better prepare students to care for SGM patients. Enhanced curricula should include helping students recognize gaps in science; make medical management decisions when clinical evidence is lacking; tailor physical exams; identify community resources for SGM patient support; understand legal context for SGM patients; address lesbian-specific health concerns; and address SGM subpopulations’ needs for health screening and medical management.

## List of Abbreviations

AAMC: Association of American Medical Colleges

GW: The George Washington University

LGBTQI: Lesbian, gay, bisexual, transgender, queer, and intersex

SGM: Sexual and gender minorities
